# Chicken miR-126-5p negatively regulates antiviral innate immunity by targeting TRAF3

**DOI:** 10.1186/s13567-022-01098-x

**Published:** 2022-10-12

**Authors:** Jie Wang, Yuqiang Cheng, Longlong Wang, Aixi Sun, Zhenyu Lin, Wenxian Zhu, Zhaofei Wang, Jingjiao Ma, Henan Wang, Yaxian Yan, Jianhe Sun

**Affiliations:** 1 Shanghai Key Laboratory of Veterinary Biotechnology, Key Laboratory of Urban Agriculture (South), Ministry of Agriculture, School of Agriculture and Biology, Shanghai Jiao Tong University, Shanghai, China; 2 Shanghai Yuan Song Biotechnology Co., LTD., Shanghai, China

**Keywords:** Chicken, miR-126-5p, TRAF3, RNA viruses, innate immunity

## Abstract

Innate immunity plays an essential role in preventing the invasion of pathogenic microorganisms. However, innate immunity is a double-edged sword, whose excessive activation is detrimental to immune homeostasis and even leads to a “cytokine storm” of the infected host. The host develops a series of negative regulatory mechanisms to balance the immune response. Here, we report a negative regulatory mechanism of chicken innate immunity mediated by miRNA. In the GEO database, we found that miR-126-5p was markedly up-regulated in chickens infected by RNA viruses. Upregulation of miR-126-5p by RNA virus was then further shown via both a cell model and in vivo tests. Overexpression of miR-126-5p significantly inhibited the expression of interferon and inflammatory cytokine-related genes induced by RNA viruses. The opposite result was achieved after the knockdown of miR-126-5p expression. Bioinformatics analysis identified TRAF3 as candidate target gene of miR-126-5p. Experimentally, miR-126-5p can target TRAF3, as shown by the effects of miR-126-5p on the endogenous expression of TRAF3, and by the TRAF3 3'UTR driven luciferase reporter assay. Furthermore, we demonstrated that miR-126-5p negatively regulated innate immunity by blocking the MAVS-TRAF3-TBK1 axis, with a co-expression assay. Overall, our results suggest that miR-126-5p is involved in the negative regulation of chicken innate immunity, which might contribute to maintaining immune balance.

**Supplementary Information:**

The online version contains supplementary material available at 10.1186/s13567-022-01098-x.

## Introduction

The innate immunity system is the first line of host defense against pathogenic microorganisms. During viral or bacterial infections, the innate immune system through a series of pattern-recognition receptors (PRR), including Toll-like receptors (TLR), retinoic acid-inducible gene I (RIG-I)-Like receptors (RLR), and the nucleotide-binding oligomerization domain (NOD)-like receptor family proteins (NLR), recognize specific pathogen-associated molecular patterns (PAMP) and cause the release of type I interferon (IFN) and inflammatory cytokines, triggering antiviral innate immune responses [1, 2].

RNA viruses such as influenza A virus (IAV), avian influenza virus (AIV), and Newcastle disease virus (NDV) cause serious diseases in humans and animals, especially chickens, which have a high mortality rate after infection [3]. TRL, RLR, and NLR are the three important influenza viruses host PRR. They are distributed on the surface and in the cytoplasm of cells. RLR include RIG-I, melanoma differentiation-associated gene 5 (MDA5), and laboratory of genetics and physiology 2 (LGP2) [4]. However, chickens lack RIG-I, and MAD5 plays a more important role in recognizing RNA viruses than mammalian MDA5 [5, 6]. In mammals, during virus infection, RIG-I and MAD5 interact with virus RNA and recruit tumor necrosis factor receptor-associated factor 3 (TRAF3) through the adaptor protein MAVS. TRAF3 is also an adaptor protein that phosphorylates TANK-binding kinase 1 (TBK1) and an inhibitor of nuclear factor-κB (IκB) kinase (IKK) to dephosphorylate the transcription factor IRF3 into the nucleus to activate the production of type I interferon [7, 8]. In birds, IRF3 is also missing, but they have IRF7, and the structure of avian IRF7 is similar to mammalian IRF3, and it also activates the secretion of type I IFN [9, 10].

Viral invasion causes the release of a series of pro-inflammatory cytokines including IL-6, IL-8, IL-1β, TNF-α, and IFN. Among them, IL-1β activates the release of IL-6, IL-8, and other pro-inflammatory cytokines, and plays an important role in host immunity and epidemic prevention [11, 12]. However, the sudden and sharp increase in circulating levels of various cytokines, leads to a “cytokine storm”, which is utilized to control early acute infections and induce antigen-specific immune responses to viral infections, but it may cause tissue damage and even death in patients [13, 14].

MiRNA are small non-coding RNA that regulate gene expression by directly binding to the mRNA 3’UTR and inhibiting the translation of mRNA [15]. Studies have demonstrated that miRNA have unique expression profiles in innate immune and adaptive immune cells, such as monocytes, macrophages, dendritic cells (DC), T cells, and B cells. Moreover, miRNA also play a key role in the regulation of host immunity and “cytokine storms” [16–18]. MicroRNA have been identified as key players in virus-host interaction, and evidence shows that miRNA can also affect the replication and pathogenesis of RNA viruses by directly binding to the RNA virus genome or through virus-mediated changes in the host transcriptome [19–21]. Generally, during viral infection, viral proteins inhibit pattern recognition receptor recognition or downstream signal cascades to prevent the production of ISG. However, it has recently been proposed that viruses mediate the regulation of IFN signaling cascades by altering host miRNA levels.

In this study, we analyzed the transcriptome data (GSE111868) of chickens infected with NDV and AIV and found that miR-126-5p is a differentially expressed miRNA AIV and NDV infection of chicken DF1 cells significantly increases gga-miR-126-5p expression, and miR-126-5p in chicken DF1 cells inhibits innate immune and inflammatory cytokine related gene expression, and promotes RNA virus replication by targeting TRAF3. In addition, overexpression of chTRAF3 significantly activates the innate immunity of chickens. In conclusion, we found that miR-126-5p, by blocking the MAVS-TRAF3-TBK1 axis, negatively regulates chicken innate immunity and promotes RNA virus replication.

## Materials and methods

### Cell culture and virus

The chicken embryonic fibroblast cell line DF1 and human 293 T cells were obtained from ATCC and cultured in DMEM supplemented with 10% FBS; the cells were incubated at 37 ℃ in a 5% CO_2_ incubator. The low virulent strain LaSota green fluorescent protein (GFP) tagged NDV was isolated from chickens on a farm in the Shandong province, China. The GFP tagged vesicular stomatitis virus (VSV) VSV-GFP was stored in our laboratory [22]. AVI virus was a A/Chicken/Shanghai/010/2008 (H9N2) (SH010) isolate from chicken in Shanghai, China, in 2008 and identified as the H9N2 avian influenza A virus. The viruses were purified, propagated, and stored as described in our previous study [23].

### Animals and treatment

Eighteen 3‐week‐old SPF chickens were purchased from the Shanghai Academy of Agricultural Sciences. All chickens were kept in negative-pressure-filtered air isolators and fed as recommended. After one week of adaptation, eighteen SPF chickens were randomly divided into three groups: the control group (control); the NDV-infected group and the AIV-infected group. The virus was diluted with phosphate buffer solution (PBS) and inoculated with eye and nose drops. The AIV group was inoculated with 10^4^ EID_50_ AIV viruses, the NDV group was inoculated with 10^5^ EID_50_ NDV viruses, and the control group was inoculated with 200 µL PBS. Three days after the inoculation, the chickens in each group were stunned with ether and killed. The spleens, livers, kidneys, tracheas, and bursa of Fabricius of these animals were collected, then immediately frozen in liquid nitrogen and stored at −80 °C for later use.

### Construction of plasmid

Based on the chicken TRAF3 sequence (NC_006092.5) obtained from the National Center for Biotechnology Information (NCBI), TRAF3-F and TRAF3-R primers were designed and used to amplify TRAF3 from DF1 cells cDNA. The PCR product was ligated into a pTOPO-Blunt vector (Aidlab, Beijing, China) and the positive colonies were sent to the Beijing Genomics Institute (Beijing, China) for sequencing. Then the pcDNA3.1-TRAF3 plasmids were constructed by inserting full-length TRAF3 into the Hind III and EcoR I sites of the pcDNA3.1 expression vector using a Hieff Clone® Plus One Step Cloning Kit (Yeasen, Shanghai, China). The TRAF3 3’ UTR sequences were cloned from the DF1 gDNA, which includes the binding sites of miR-126-5p predicted by TargetScan. In addition, the TRAF3 3’ UTR binding site mutant sequences were cloned based on the TRAF3 3’ UTR sequences, then TRAF3 3’ UTR wild type or mutant sequences were inserted into the pmiR-GLO plasmid, respectively. The DH5α chemically competent cell (Tsingke Biology Technology, Beijing, China) was used for plasmid transformation.

The primer sequence is as follows:

TRAF3

Forward primer: TAGTCCAGTGTGGTGGAATTCATGGACACCAGTAAGAAGACA.

Reverse primer: AACGGGCCCTCTAGACTCGAGTCAGGGGTCTGGTAGATCCGA.

TRAF3-3’UTR

Forward primer: GAGCTCGCTAGCCTCGAGAGGATTTTTGTTTTGTTCTGTT.

Reverse primer: CTGCAGGTCGACTCTAGATTTCTAAAGAGAAATAACAGAA.

TRAF3-3’UTR- mutant.

Forward primer: GTTTCGTGTTCTGCTTTGTAAGAAGATCTTGGA.

Reverse primer: AGCAGAACACGAAACAGAGACCAGATGAGGCCTTA.

### Cell transfection

DF1 cells were seeded in 12-well or 6-well plates (NEST Biotechnology, Wuxi, China) at 5 × 10^5^/mL or 1 × 10^6^/mL and transfected with miR-126-5p mimics or mimics control (NC) at 100 nmol/mL. miR-126-5p inhibitor or inhibitor control (NA) at 200 nmol/mL (GenePharma, Shanghai, China) was performed with Nulen PlusTrans™ Transfection Reagent (Yeasen, Shanghai, China) according to the manufacturer’s protocol. The pcDNA3.1 vector or TRAF3 plasmid were transfected at 500 ng/well in 12-well plates or at 1000 ng/well in 6-well plates with transfection Reagent. Forty-eight hours after transfection, the cells were used for the subsequent experiments. The sequences of miR-126-5p mimics and inhibitors were the following:

NC sense (5’- 3’): UUCUCCGAACGUCUCACG.

NC antisense (5’- 3’): ACGUGACACGUUCGGAGAATT.

Gga-miR-126-5p mimics (5’- 3’):

AUUAUUACUUUUGGUACGCGCGUACCAAAAGUAAUAAUGUU

Inhibitor NC (5’- 3’): CAGUACUUUUGUGUAGUACAA.

Gga-miR-126-5p inhibitor (5’- 3’): CGCGUACCAAAAGUAAUAAUG.

### Bioinformatics Screening and miR-126-5p Target Gene Prediction

The RNA virus infection chicken RNA-seq dataset GSE111868 was initially downloaded from the NCBI GEO portal. We systematically analyzed differentially expressed miRNA (DEG) of NDV F48E9, and LaSota infected chicken embryos using the R package limma. |Log fold change (FC)|> 2 and a *p* value < 0.05 were set as thresholds to screen out the DEG as described in our previous study [24]. The Ven Maps, Heat Maps, and Volcano Maps analyses were performed using the Sangerbox tools, a free online platform for data analysis. The target genes of miR-126-5p were predicted using the online website TargetScan, miR-126-5p (miRNA name), and *Gallus gallus* (organism) were chosen [25]. The Kyoto Encyclopedia of Genes and Genomes (KEGG) and Gene Ontology (GO) analysis of the target gene of miR‐126‐5p were used on the online website KOBAS 3.0 [26].

### Protein–protein interaction network analysis

The protein–protein interaction (PPI) network was performed using STRING. TRAF3 (protein name) and *Gallus gallus* (organism) were chosen, as described in [27].

### RNA extraction and qPCR

Total RNA was extracted from the cells with AG RNAex Pro Reagent (Accurate Biology, Hunan, China). mRNA was reverse‐transcribed with reverse transcription kits (Accurate Biology, Hunan, China), and the cDNA using the SYBR green PCR mix (Tsingke Biology Technology, Beijing, China) with the Applied Biosystems machine (ABI 7500; Thermo Fisher Scientific). Relative gene expression was analyzed using the 2^−ΔΔCt^ method. The β‐actin and U6 small RNA were the internal references when examining the levels of genes and miR‐126‐5p. The primer sequences for the genes are shown in Additional file 1.

### Dual-luciferase reporter assays

293 T cells were seeded in 24-well plates at 2.5 × 10^5^/mL. The pmiR-TRAF3-WT-GLO or mutant pmiR-TRAF3-mutant-GLO plasmid (250 ng/well) were co-transfected with miR-126-5p mimics or NC (100 nmol/mL) into 293T cells. At 48 h post-transfection, luciferase activities were measured using the dual-luciferase reporter assay system (Promega, USA) according to the manufacturer’s protocol.

### Statistical analysis

The results are expressed as the mean ± SD. GraphPad Prism 8.0 was utilized to graph the results. Data were analyzed by the Student’s *t* test. *p* < 0.05 was considered statistically significant, and *p* < 0.01 was considered highly statistically significant (*p < 0.05; **p < 0.01).

## Results

### The expression of miR-126-5p is upregulated by RNA virus in chickens

**Figure 1:**
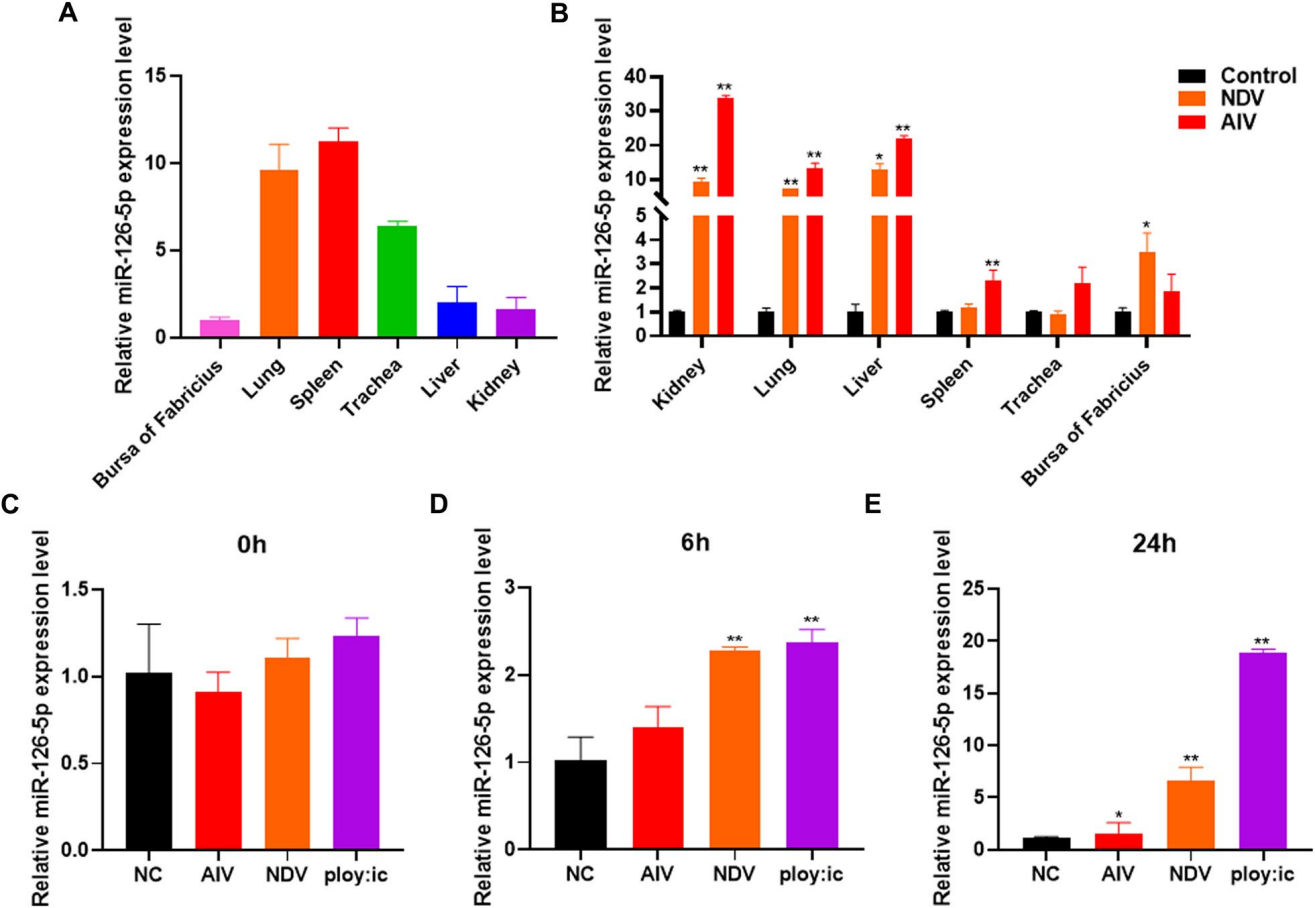
**RNA viruses upregulate the expression of miR-126-5p in chickens.** **A** qPCR analysis of miR-126-5p expression in the bursa of Fabricius, lungs, spleens, tracheas, livers, and kidneys of healthy chickens. **B** The changes of miR-126-5p expression in the bursa of Fabricius, lungs, spleens, tracheas, livers, and kidneys of chickens after infection with NDV and AIV by qPCR. **C**-**E** DF1 cells were infected with AIV or NDV or poly (I: C), and the expression of miR-126-5p at 0 h (**C**), 6 h (**D**), and 24 h (**E**) post-infection was detected by qPCR. The data are expressed as the mean ± SD; *n* = 3. *p < 0.05, **p < 0.01.

miRNA are a kind of small non-coding RNA that regulate many biological processes in cells. A large number of studies have demonstrated that miRNA also influence virus replication. To explore the miRNA that affect virus replication, we analyzed the GEO (GSE111868) RNA-seq dataset and previous research results [28] on chickens infected with NDV AIV and found that miR-126-5p was significantly up-regulated (Additional file 2). The expression level of the miRNA is closely related to its function. To explore the role of miR-126-5p in the process of virus invasion, we tested the expression of miR-126-5p in different tissues of chickens and found that the expression of miR-126-5p was the highest in the spleen of chickens and the lowest in the bursa of Fabricius (Figure 1A). The spleen is an immune organ of the body, and miR-126-5p has a high expression level, indicating that miR-126-5p may be related to the regulation of the immune responses of chickens. Next, we tested the expression of miR-126-5p in different tissues after chickens were infected with NDV and AIV. It was noted that both NDV and AIV infection significantly increased the expression of miR-126-5p (Figure 1B). To further explore the changes of miR-126-5p in the process of RNA virus invasion, we used AIV, NDV, and the double-stranded RNA (dsRNA) analogue poly (I: C) which is a simulative stimulus of virus RNA, to infect chicken DF1 cells. Cells at different time points of infection were collected to detect the expression of miR-126-5p, it was found that NDV and poly (I: C) significantly increased the expression of miR-126-5p after 6 h of infection, while AIV, NDV, and poly (I: C) significantly increased the expression of miR-126-5p after 24 h of infection (Figures 1B–D). These results suggest that miR-126-5p may be involved in the regulation of host responses to RNA virus infection or RNA virus PAMP.

### Bioinformatics analyses indicate that miR-126-5p is involved in the regulation of innate immunity

**Figure 2:**
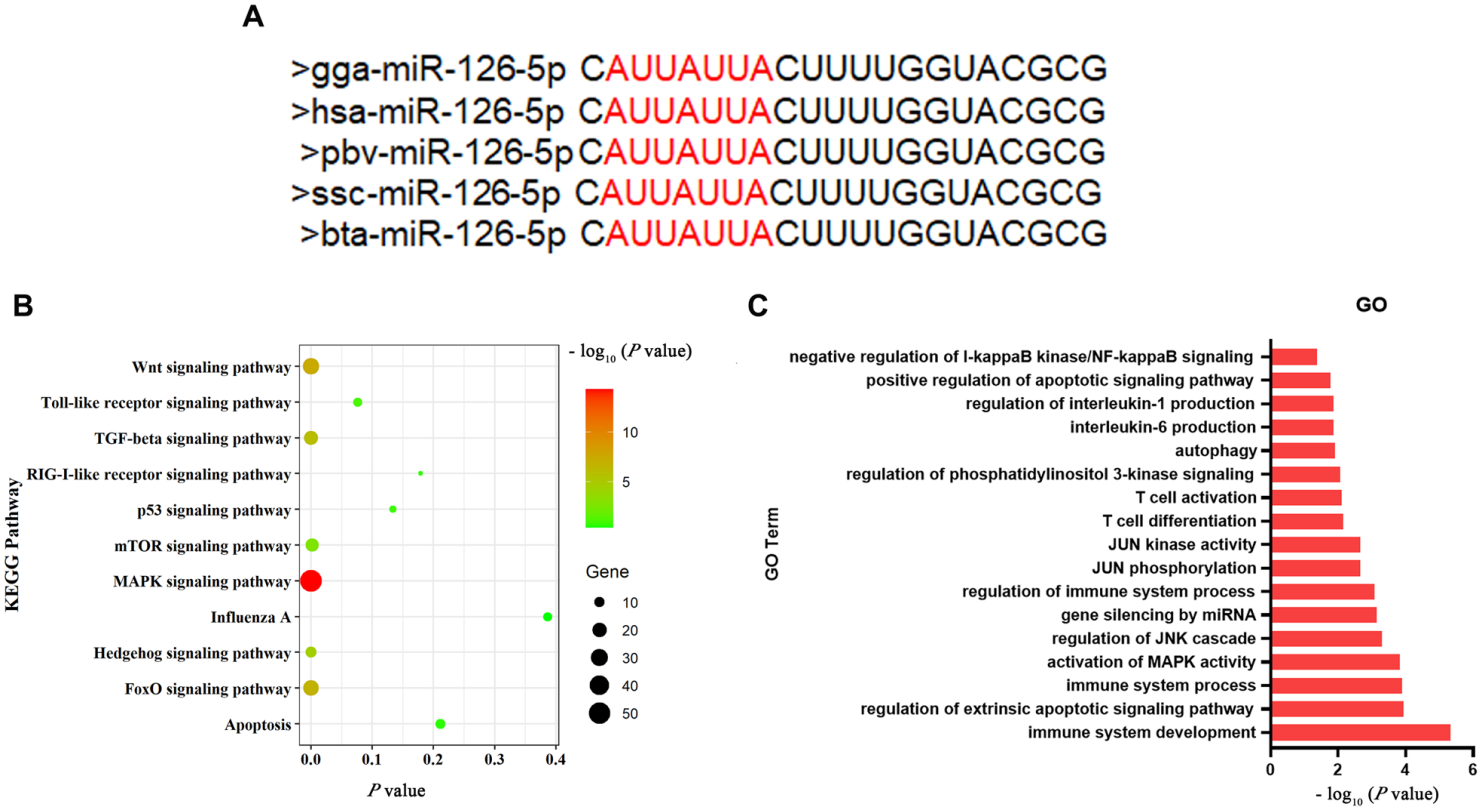
**Bioinformatics analyses indicate miR-126-5p is involved in the regulation of innate immunity.** **A** Sequence alignment of miR-126-5p from various vertebrate species. **B** KEGG pathway analysis of miR-126-5p target genes. **C** GO term analysis of miR-126-5p target genes.

To investigate the function of miR-126-5p in chicken innate immunity, we thoroughly analyzed the mature seed sequence of miR-126-5p. It was found that miR-126-5p was widely present in different species such as humans, pigs, cattle, chickens, etc. (Figure 2A). In addition, KEGG pathways and GO analyses of the target genes of miR‐126‐5p revealed that it was involved in the regulation of multiple innate immune-related signaling pathways, such as the TLR and RIG-I-like receptor signaling pathways (Figure 2B). GO analysis unveiled that the majority of genes participated in regulating immune cell differentiation and inflammatory factor secretion (Figure 2C). These results imply that miR-126-5p has notable effects on chicken innate immunity.

**Figure 3:**
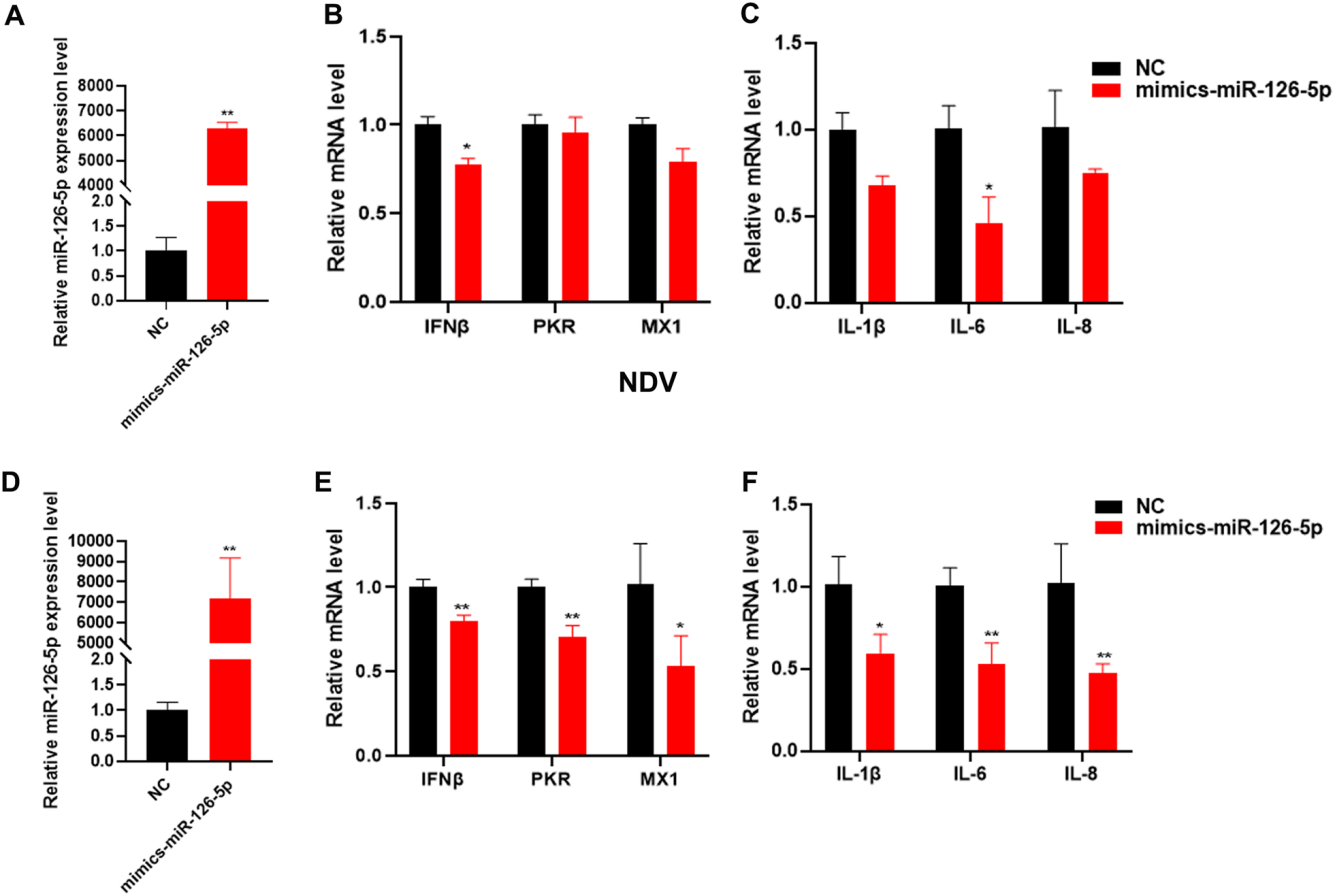
**Overexpression of miR-126-5p inhibits chicken antiviral innate immunity during NDV infection.** **A** qPCR analysis of miR‐126‐5p overexpression efficiency, after miR-126-5p mimics or NC (Negative control) transfection of DF1 cells at 24 h. **B** qPCR was used to detect the RNA levels of innate immune-related genes, including IFNβ, PKR, and MX1, after overexpression of miR-126-5p. **C** qPCR detection of inflammatory cytokine-related gene RNA levels, such as IL-Iβ, IL-6, and IL-8 expression after overexpression of miR-126-5p. **D**, **E** Transfection of DF1 cells with miR-126-5p mimics or NC, followed by infection with by NDV for 12 h. **D** qPCR analysis of miR‐126‐5p overexpression efficiency. **E** qPCR detection of IFNβ, PKR and MX1 expression levels. **F** qPCR detection of inflammatory cytokine-related gene IL-Iβ, IL-6 and IL-8 levels. The data are expressed as the mean ± SD; *n* = 3. *p < 0.05, **p < 0.01.

### Overexpression of miR-126-5p inhibits chicken antiviral innate immunity during NDV infection

To ﻿verify whether miR-126-5p participates in the regulation of chicken innate immunity, we transfected DF1 cells with miR-126-5p mimics or NC and analyzed the expression of innate immune-related IFNβ genes, eukaryotic translation initiation factor 2 alpha kinase 2 (PKR), MX1–myxovirus (influenza virus) resistance 1 (MX1), inflammatory cytokine related genes interleukin 1β (IL-1β), interleukin 6 (IL-6) and Interleukin-8 (IL-8). Overexpression of miR-126-5p was confirmed by qPCR (Figure 3A). We found that overexpression of miR-126-5p inhibited the expression of the above genes, but only significantly reduced IFNβ, and IL-6 expression (Figures 3B, C) while transfecting DF1 cells with miR-126-5p mimics or NC, followed by infection with NDV for 12 h (Figure 3D), showed that overexpression of miR-126-5p markedly inhibited innate immunity and inflammatory cytokine related genes expression (Figures 3E and F). These results indicate that the RNA virus used miR-126-5p to evade the host’s antiviral innate immune response.

### Inhibition of miR-126-5p promotes chicken antiviral innate immunity during NDV infection

**Figure 4:**
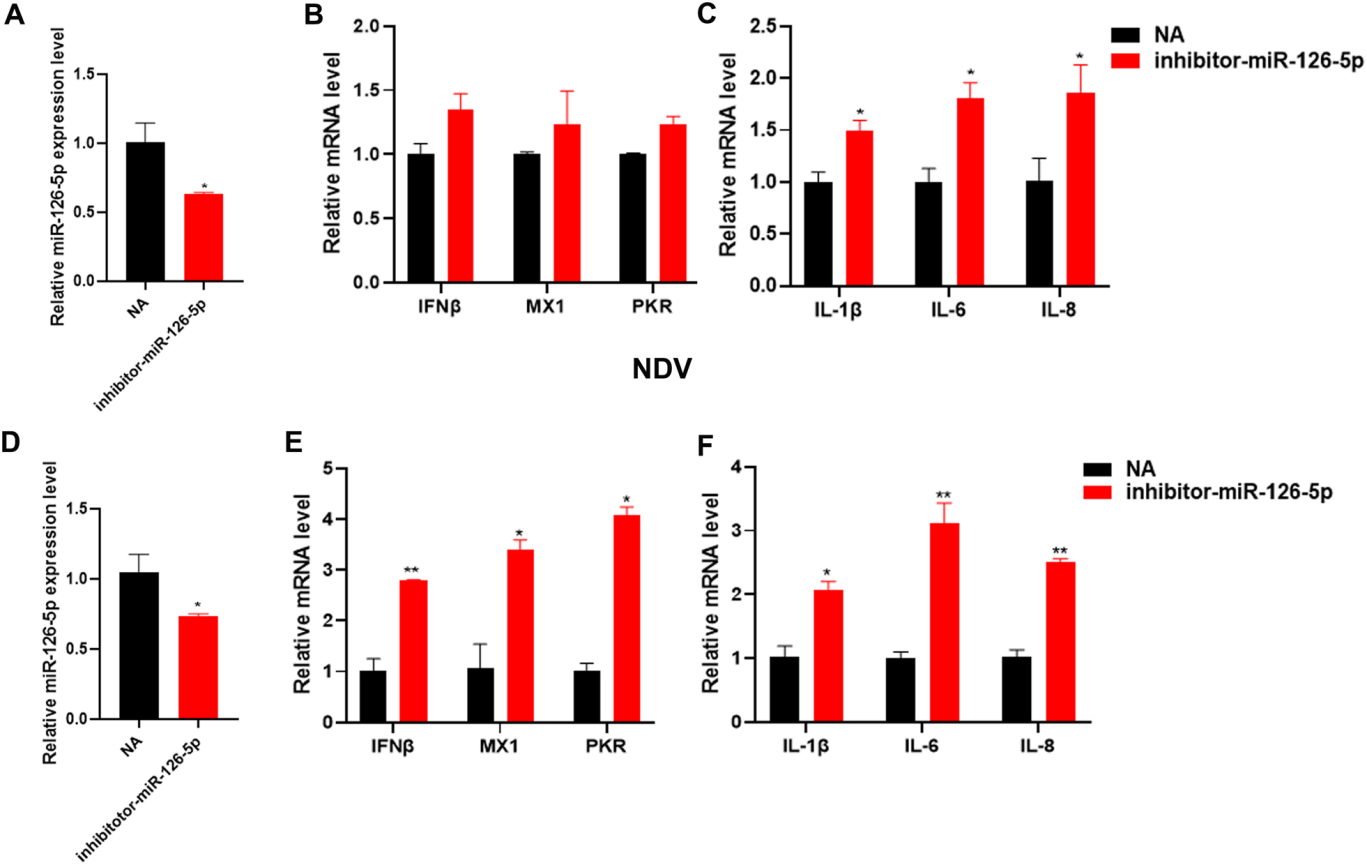
**Inhibition of miR-126-5p promoted chicken antiviral innate immunity during NDV infection.** **A** qPCR analysis of miR‐126‐5p inhibition efficiency, after miR-126-5p inhibitor or NA (Negative control) transfection of DF1 cells at 24 h. **B** qPCR detection of the RNA levels of innate immune-related genes, including IFNβ, PKR, and MX1 after inhibition of miR-126-5p. **C** qPCR detection of inflammatory cytokine-related gene mRNA expression, such as IL-Iβ, IL-6, and IL-8 after the inhibition of miR-126-5p. **D**, **E** Transfected DF1 cells with miR-126-5p inhibitor or NA, followed by infection NDV for 12 h. **D** qPCR analysis of miR‐126‐5p inhibition efficiency. **E** qPCR detection of IFNβ, PKR and MX1 expression levels. **F** qPCR detection of the expression levels of the inflammatory cytokine-related genes IL-Iβ, IL-6 and IL-8. The data are expressed as the mean ± SD; *n* = 3. *p < 0.05, **p < 0.01.

Overexpression of miR-126-5p inhibits the expression of chicken antiviral innate immunity-related genes. To further study the role of miR-126-5p in chicken antiviral innate immunity, we transfected DF1 cells with miR-126-5p inhibitor or NA. It was found that miR-126-5p inhibitor significantly inhibited the expression of miR-126-5p (Figure 4A). Inhibition of miR-126-5p increased the expression of IFNβ, MX1, and PKR (Figure 4B), and obviously increased the expression of inflammatory cytokine related genes IL-1β, IL-6, and IL-8 (Figure 4C). Similarly, inhibiting the expression of miR-126-5p infection with NDV significantly promoted the expression of IFNβ, PKR, MX1, IL-1β, IL-6, and IL-8 (Figures 4D–F). These show that inhibition of miR-126-5p promotes chicken antiviral innate immunity after RNA virus invasion. These results also point out that miR-126-5p participates in regulating chicken antiviral innate immunity.

### The interferon signaling pathway has no role in regulating miR-126-5p expression

**Figure 5:**
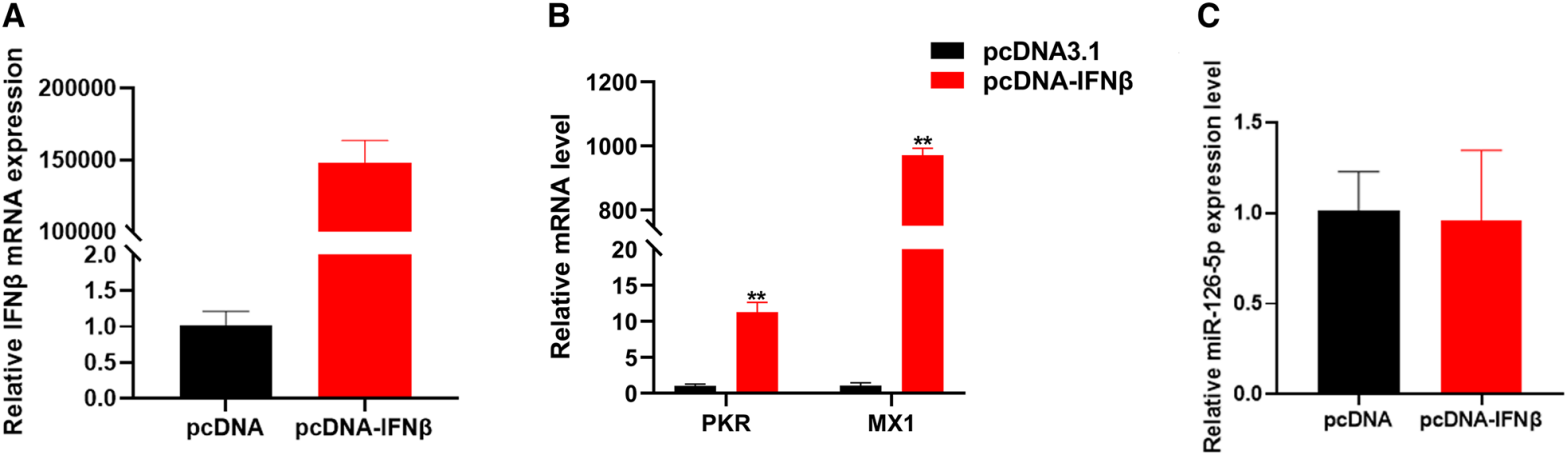
**The interferon signaling pathway has no role in the regulation of miR-126-5p expression.** **A** After transfection of pcDNA-IFNβ into DF1 cells, the overexpression efficiency of IFNβ was detected by qPCR. **B** qPCR detection of the expression of PKR and MX1 after overexpression of IFNβ. **C** qPCR detection of the expression of miR-126-5p after overexpression of IFNβ. The data are expressed as the mean ± SD; *n* = 5. *p < 0.05, **p < 0.01.

After the virus infects the host, it activates the interferon signaling pathway and promotes ISG in response to virus invasion. In this study, we found that virus invasion significantly increased the expression of miR-126-5p, and KEGG pathway analysis also found that miR-126-5p participates in the regulation of the type I interferon signaling pathway (Figure 2B). These indicate that miR-126-5p may be an interferon-stimulated gene. To demonstrate this, we overexpressed chicken IFNβ in DF1 cells (Figure 5A), and found that overexpression of IFNβ significantly increased the expression of ISG, such as PKR and MX1 (Figure 5B), but did not affect the expression of miR-126-5p (Figure 5C). This means that the interferon signaling pathway could not regulate the expression of miR-126-5p, and miR-126-5p was not an ISG.

### MiR-126-5p promotes RNA virus replication

**Figure 6:**
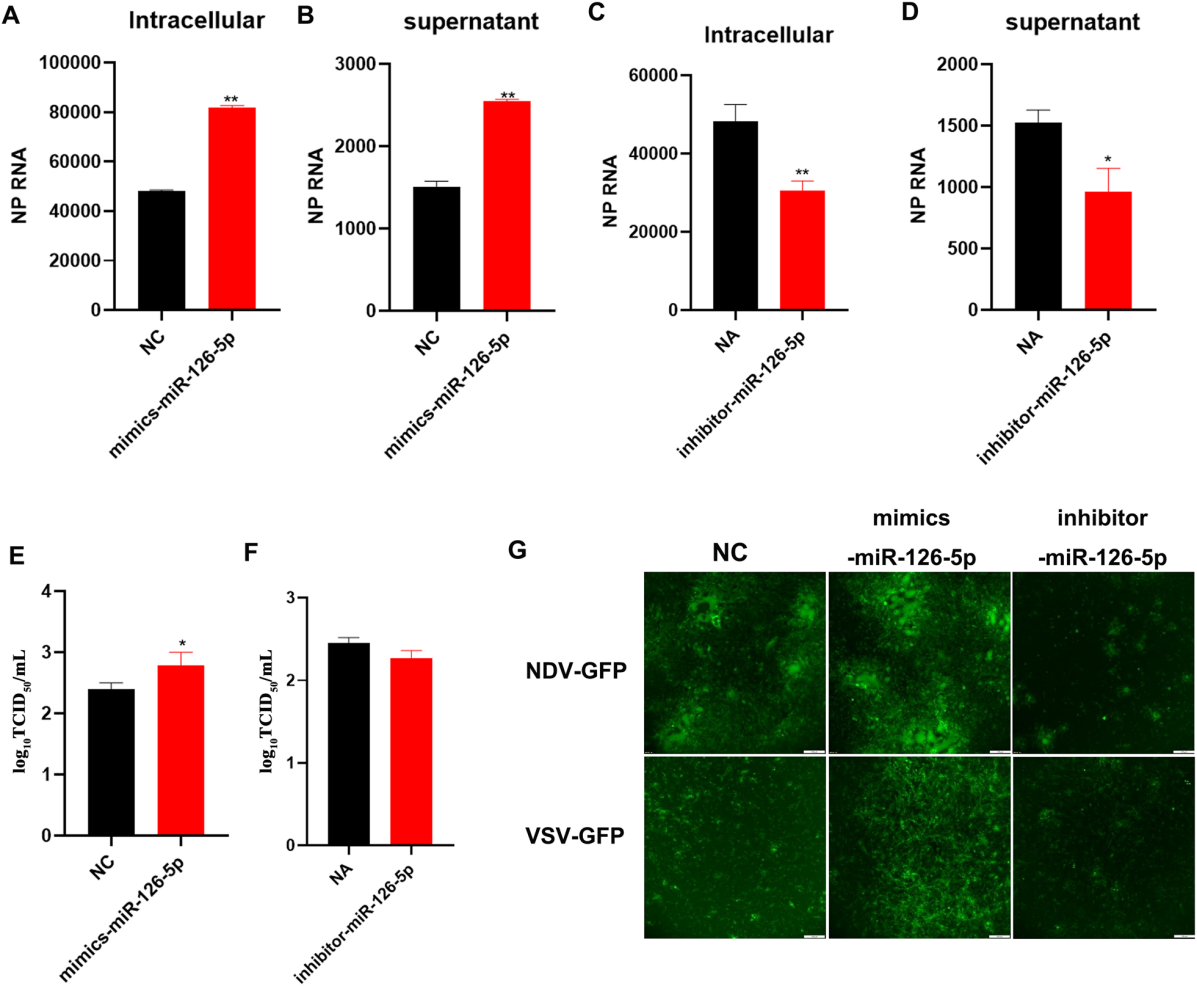
**miR126-5p promotes RNA virus replication.** **A**, **B** DF1 cells were transfected with miR-126-5p mimics or NC, followed by infection with NDV at MOI = 0.01 after 6 h, and intracellular and supernatant relative levels of NP RNA were measured by absolute quantitative real-time PCR detection. **C**, **D** DF1 cells were transfected with miR-126-5p inhibitor or NA, followed by infection with NDV at an MOI of 0.01. After 6 h, intracellular and supernatant relative levels of NP RNA were measured by absolute quantitative real-time PCR detection. **E**, **F** The effect of miR-126-5p on NDV replication by TCID_50_. **G** NDV-GFP or VSV-GFP infected DF1 cells were visualized 48 h post-transfection and post-infection using fluorescence microscopy and representative images are shown for NC, miR-126-5p mimics or inhibitor transfected cells. Scale bar = 100 μm. The data are expressed as the mean ± SD; n = 3. *p < 0.05, **p < 0.01.

Immune-related genes are essential for regulating virus replication. In this study, we found that miR-126-5p regulates the expression of immune-related genes. To investigate the regulatory role of miR-126-5p in NDV replication, we transfected miR-126-5p mimics or inhibitors into DF1 cells, before infecting the cells with NDV at a multiplicity of infection 0.01 MOI. Then the copy number of the virus in the cells and culture medium were determined by detecting the expression of the NDV NP gene. The expression of NDV NP RNA as a sign of virus replication was significantly increased in the intracellular and supernatant after overexpression of miR-126-5p (Figures 6A and B), while inhibiting the expression of miR-126-5p obviously decreased the NDV NP RNA expression (Figures 6C and D). At the same time, we used the above supernatant to transfect DF1 cells with the miR-126-5p mimics or inhibitor, followed by infection with NDV for measurement of viral load by standard assays, such as 50% tissue culture infective dose (TCID_50_). Overexpression of miR-126-5p markedly increased the NDV viral titer and inhibition of miR-126-5p reduced the viral titer as determined using the TCID_50_ assay (Figures 6E, F). This result shows that overexpression of miR-126-5p promotes NDV replication, while inhibition of miR-125-5p inhibits NDV replication. To further confirm these results, we infected DF1 cells with NDV-GFP and VSV-GFP after overexpression or inhibition of miR-126-5p and found that overexpression of miR-126-5p increased the fluorescence intensity while inhibiting miR-126-5p decreased the fluorescence intensity (Figure 6G). This suggests that miR-126-5p promotes RNA virus replication.

### MiR-126-5p inhibits chicken antiviral innate immunity by targeting TRAF3

**Figure 7:**
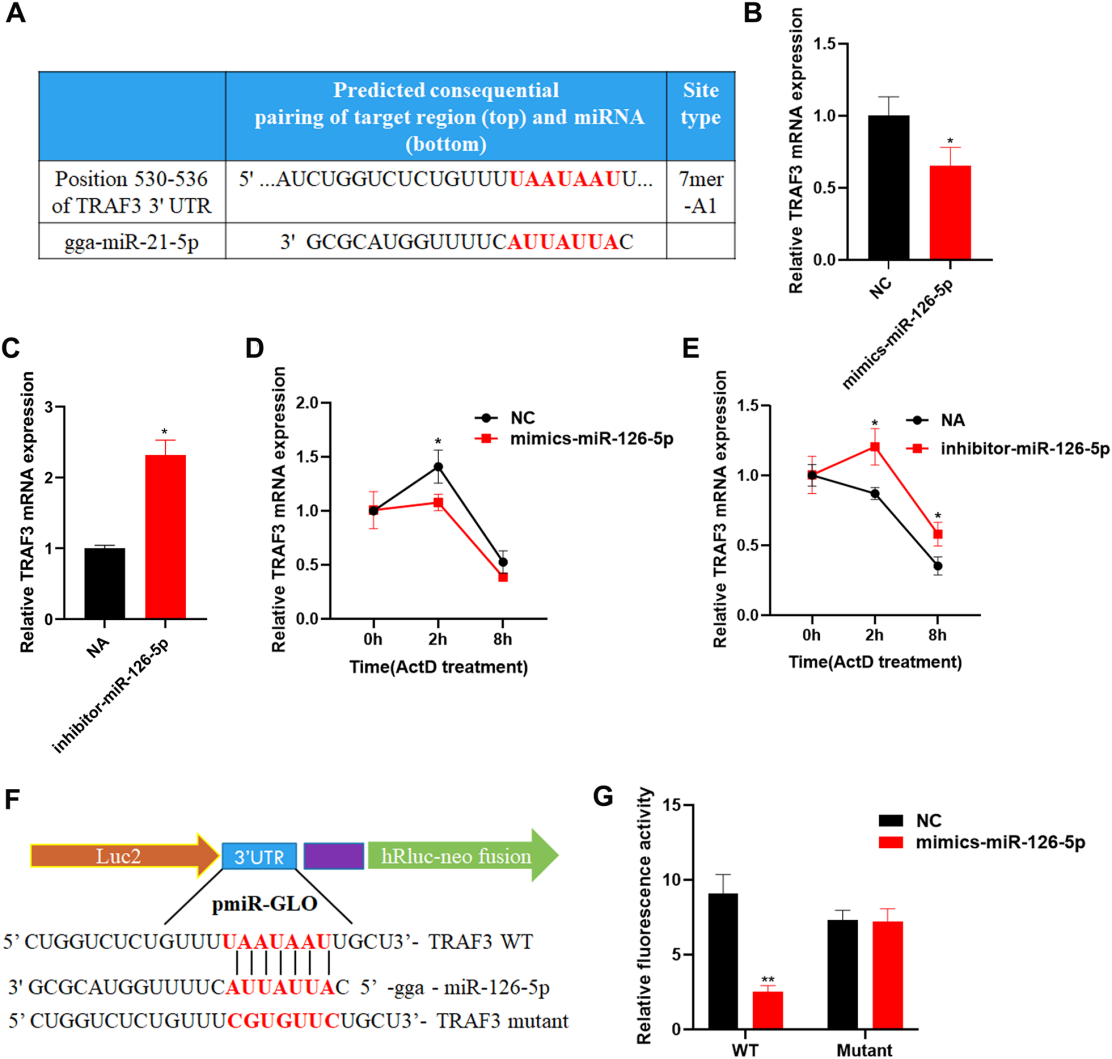
**miR-126-5p inhibits chicken antiviral immune responses by targeting TRAF3.** **A** miR-126-5p and TRAF3 binding site prediction. **B** qPCR detection of the expression of TRAF3 after over-expression of miR-126-5p. **C** qPCR detection of the expression of TRAF3 after inhibition of miR-126-5p. qPCR analysis of the expression of TRAF3 in DF1 cells that were overexpressed (**D**) or inhibited (**E**) miR-126-5p 12 h, infected with NDV for 24 h at an MOI of 0.01, and then treated with actinomycin D (ActD; 10 g/mL) for the indicated times. **F** The TRAF3 3'UTR containing the miR-126-5p binding sequence or mutant sequence genes was cloned into the pmiR-GLO vector as shown. **G** HEK293T cells were transfected with pmiR-TRAF3-WT-GLO or mutant pmiR-TRAF3-mutant-GLO along with miR-126-5p mimics or NC, lysed after 24 h, and subjected to luciferase assay. The data are expressed as the mean ± SD; *n* = 3. *p < 0.05, **p < 0.01.

MiRNA exert a regulatory function by binding target gene mRNA 3'UTRs to inhibit the translation of target genes. To understand the molecular mechanism of miR-126-5p in the regulation of chicken antiviral immune responses during RNA virus infection, we used the online target gene prediction software TargetScan and found that TRAF3 was a candidate target gene for miR-126-5p (Figure 7A). Overexpression of miR-126-5p significantly inhibited the expression of TRAF3 (Figure 7B), while inhibition of miR-126-5p radically increased the expression of TRAF3 (Figure 7C), indicating that miR-126-5p has a regulatory relationship with TRAF3. MiRNA bind to target genes to inhibit their translation, thereby accelerating the degradation of target genes. To verify whether miR-126-5p accelerates TRAF3 degradation, we overexpressed or inhibited the expression of miR-126-5p; the cells were treated with actinomycin D, an inhibitor of transcription, and the cells were collected after being treated for 0, 2, and 8 h. It was found that overexpression of miR-126-5p significantly accelerated the degradation of TRAF3 (Figure 7D), while inhibition of miR-126-5p was observed to slow down the degradation of TRAF3 (Figure 7E). These results show that miR-126-5p may target TRAF3. To further verify whether miR-126-5p targeted TRAF3 regulation of antiviral immune responses, we used the pmiR-GLO plasmid to construct a TRAF3 3'UTR containing miR-126-5p binding sequence pmiR-TRAF3-WT-GLO and mutant pmiR-TRAF3-mutant-GLO plasmids (Figure 7F). pmiR-TRAF3-WT-GLO or pmiR-TRAF3-mutant-GLO were co-transfected with miR-126-5p mimics or NC. We found that miR-126-5p mimics significantly inhibited the fluorescent activity of the pmiR-TRAF3-WT-GLO plasmid, but did not affect the pmiR-TRAF3-mutant-GLO (Figure 7G). These results indicate that miR-126-5p inhibits chicken antiviral immune responses by targeting TRAF3.

### TRAF3 regulation of chicken innate immunity

**Figure 8:**
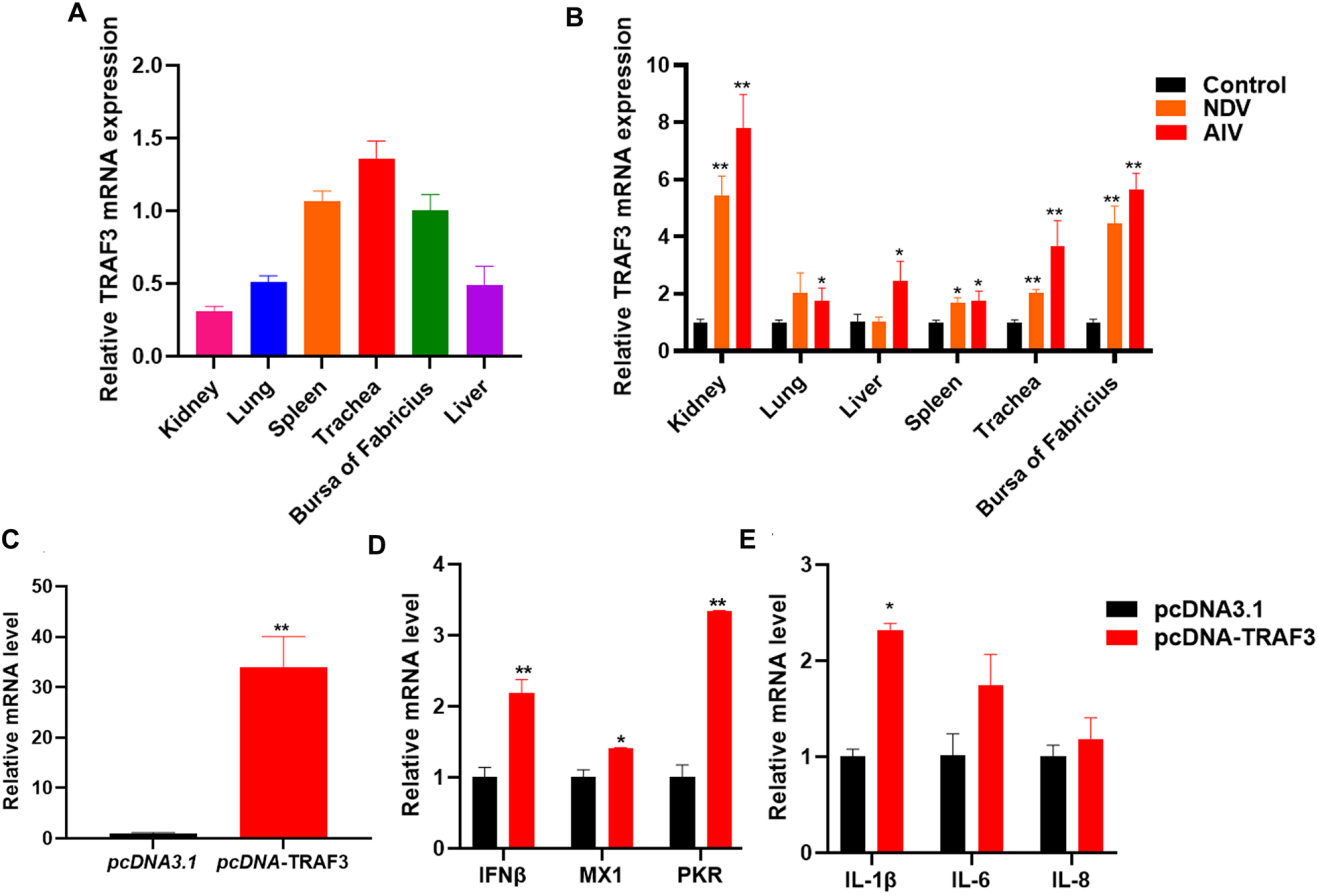
**TRAF3 regulation of chicken innate immunity.** **A** qPCR analysis of TRAF3 expression in the chicken bursa of Fabricius, lung, spleen, trachea, liver, and kidney tissues; **B** qPCR detection of the change of TRAF3 in the bursa, lung, spleen, trachea, liver, and kidney tissues after chickens were infected with NDV and AIV. **C** After transfection of pcDNA-TRAF3 into DF1 cells, the overexpression efficiency of TRAF3 was detected by qPCR. **D** qPCR detection of the expression of IFNβ, PKR and MX1 after overexpression of TRAF3. **E** qPCR detection of the expression levels of the inflammatory cytokine genes IL-Iβ, IL-6 and IL-8 after overexpression of TRAF3. The data are expressed as the mean ± SD; *n* = 3. *p < 0.05, **p < 0.01.

In order to explore the role of TRAF3 in chicken innate immunity, we detected the expression level of TRAF3 in different chicken tissues and found that TRAF3 levels in the chicken tracheas, spleens, and bursa of Fabricius were higher than those in the kidneys (Figure 8A). Chickens infected with NDV or AIV had significantly increased expression levels of TRAF3 in the above organs (Figure 8B). This shows that TRAF3 relates to chicken innate immunity. To further explore the effect of TRAF3 on chicken innate immunity, we transfected DF1 cells with pcDNA-TRAF3 or pcDNA3.1 to detect the expression levels of genes related to innate immunity (Figure 8C). TRAF3 overexpression significantly increased the expression of IFNβ and ISG genes, such as PKR and MX1 (Figure 8D), as well as the expression of inflammatory cytokine-related gene IL-1β (Figure 8E). These results suggest that TRAF3 is essential for regulating chicken antiviral innate immunity.

### MiR-126-5p negatively regulates chicken innate immunity by blocking the MAVS-TRAF3-TBK1 axis

**Figure 9:**
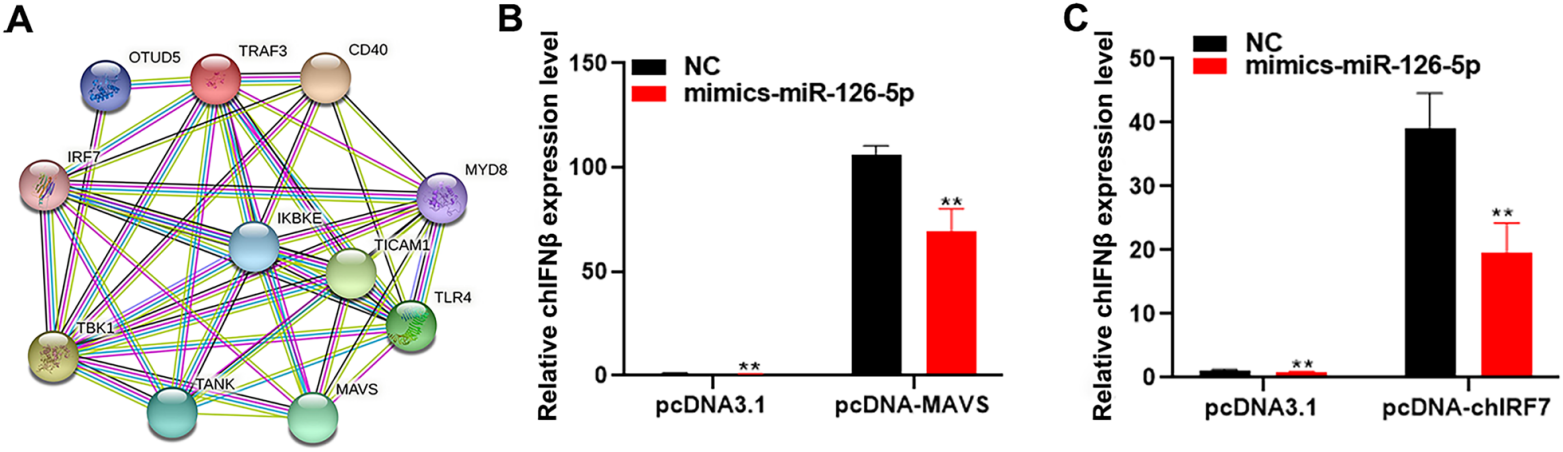
**MiR-126-5p negatively regulates chicken innate immunity by blocking the MAVS-TRAF3-TBK1 axis.** **A** TRAF3 interaction protein prediction through the online prediction software STRING. **B** qPCR detection of the expression of IFNβ after co-transfection of pcDNA-MAVS with miR-126-5p mimics or NC. **C** qPCR detection of the expression of IFNβ after co-transfection of pcDNA-IRF7 with miR-126-5p mimics or NC. The data are expressed as the mean ± SD; *n* = 3. *p < 0.05, **p < 0.01.

To better understand the underlying molecular mechanism of TRAF3-mediated chicken innate immunity, we used the online prediction software STRING to predict TRAF3 interaction proteins and found that TRAF3 interacts with many immune-related proteins such as TBK1, MAVS, and IRF7 (Figure 9A). Studies have found that TRAF3 interacts with MAVS in mammals. MAVS and IRF7 are located upstream and downstream of TRAF3, respectively. To verify whether miR-126-5p regulates chicken innate immunity by targeting TRAF3, miR-126-5p mimics were co-transfected with chMAVS or chIRF7 in the DF1 cells. We found that overexpression of miR-126-5p with MAVS or IRF7 significantly inhibited the expression of IFNβ (Figures 9C and D). These results indicate that miR-126-5p regulates chicken innate immunity by targeting TRAF3 and blocking the MAVS-TRAF3-TBK1 axis.

## Discussion

Upon virus infection, host innate immunity serves as the first line of defense against viruses. When viral RNA is released into host cells, it is recognized by host PRR and secretes type I IFNs, pro-inflammatory factors, and chemokines [29]. Type I interferon stimulates the expression of hundreds of ISG in neighboring cells and induces antiviral status [30]. In this study, we found that RNA viruses such as NDV and AIV significantly up-regulated the expression of chicken miR-126-5p. After bioinformatics analysis of the target genes of miR-126-5p, we found that miR-126-5p participates in the regulation of multiple immune-related signaling pathways. This finding indicates that miR-126-5p may be involved in chicken innate antiviral immunity. A large number of studies have concluded that miR-126-5p promotes the proliferation and migration of cancer cells such as human rectal cancer, ovarian cancer, and mouse aortic aneurysm [31–34]. Cancer is due to immune escape [35, 36], which shows that miR-126-5p is not only involved in the immunity of chickens but also participates in the immunity of mammals.

The above results indicate that miR-126-5p may be regulated by type I IFN. However, we found that chIFNβ significantly up-regulates the expression of two typical ISG, MX1 and PKR, but does not affect the expression of miR-126-5p, these show that miR-126-5p is not regulated by IFNβ. More importantly, miR-126-5p inhibits the expression of innate immunity and inflammatory cytokine-related genes following NDV infection. Similarly, EV71 infection up-regulates the expression of miR-141, resulting in down-regulation of the eukaryotic translation initiation factor 4E protein, thereby promoting EV71 replication and release [37]. These data indicate that miR-126-5p regulates host antiviral immunity. However, how the host regulates the expression of miR-126-5p after virus invasion still needs further study.

Excessive immune response after virus invasion can cause a “cytokine storm”, which severely damages host organs and even causes death [14]. A large number of studies have shown that miRNA participate in the regulation of host cytokine storms after virus invasion. For example, after influenza virus invasion, miR-302a can induce a cytokine storm, and miR-133a also has the same effect [38–40]. Interestingly, we found that miR-126-5p significantly inhibited the expression of inflammatory cytokine-related genes after NDV infection. At the same time, miR-126-5p notably promoted the replication of NDV. This indicates that miR-126-5p regulates the host's immune response by suppressing the host “cytokine storm”. This negative regulation effectively avoids damage to the host by a strong immune response.

MiRNA regulate the protein abundance of target genes by binding and inhibiting the translation of gene mRNA [15]. To study the mechanism of miR-126-5p inhibiting chicken antiviral innate immunity, we analyzed the target genes of miR-126-5p. We found that miR-126-5p targets and inhibits the expression of TRAF3. A large number of studies have shown that TRAF3 plays a significant role in the process of antiviral innate immunity [41–43]. It is a MAVS adaptor protein that undergoes k63-linked ubiquitination in mitochondria during RNA virus infection. This post-translational modification induces two kinds of Iκ-B kinase (IKK) related kinases, TANK binding kinase 1 (TBK1) and IKKi activation. These two kinases phosphorylate interferon regulatory factor 3 (IRF3) or IRF7, resulting in a type I interferon (IFN) [44, 45]. In this study, we also found that RNA virus infection in chickens significantly increases the expression of TRAF3, and overexpression of TRAF3 enhances the antiviral innate immune responses of chickens. Our previous study found that MAVS acts as a scaffold protein to recruit and phosphorylate TBK1 and IRF7 to activate IFNβ [10, 46]. Coupled with this study, we think that MAVS may first recruit TRAF3 and then recruit TBK1 and IRF7. To further demonstrate that miR-126-5p inhibits the type I interferon signaling pathway through TRAF3, we separately co-overexpressed the TRAF3 upstream and downstream proteins MAVS and IRF7 with miR-126-5p to detect the related gene expression of the type I interferon signaling pathway. It was found that the simultaneous overexpression of MAVS or IRF7 with miR-126-5p significantly inhibited the expression of IFNβ, MX1, and PKR. This shows that TRAF3 is required for the activation of type I interferon pathways.

However, in this study, we also found that miR-126-5p regulated virus replication. Existing research findings show a dual regulatory role of miRNA for virus replication [40]. First, miRNA exert an antiviral effect and inhibit virus replication by directly binding to the virus 3'NTR. In addition, miRNA directly bind to the 5'NTR of the virus to stabilize the virus structure and promote virus replication. Of course, viruses can also promote their replication by changing the abundance of host miRNA [19, 47]. Here, we found that overexpression of miR-126-5p significantly promotes virus replication, while inhibition of miR-126-5p reduces virus replication. We believe that miR-126-5p regulation of viral replication may be related to miR-126-5p negative regulation of innate immunity.

**Figure 10:**
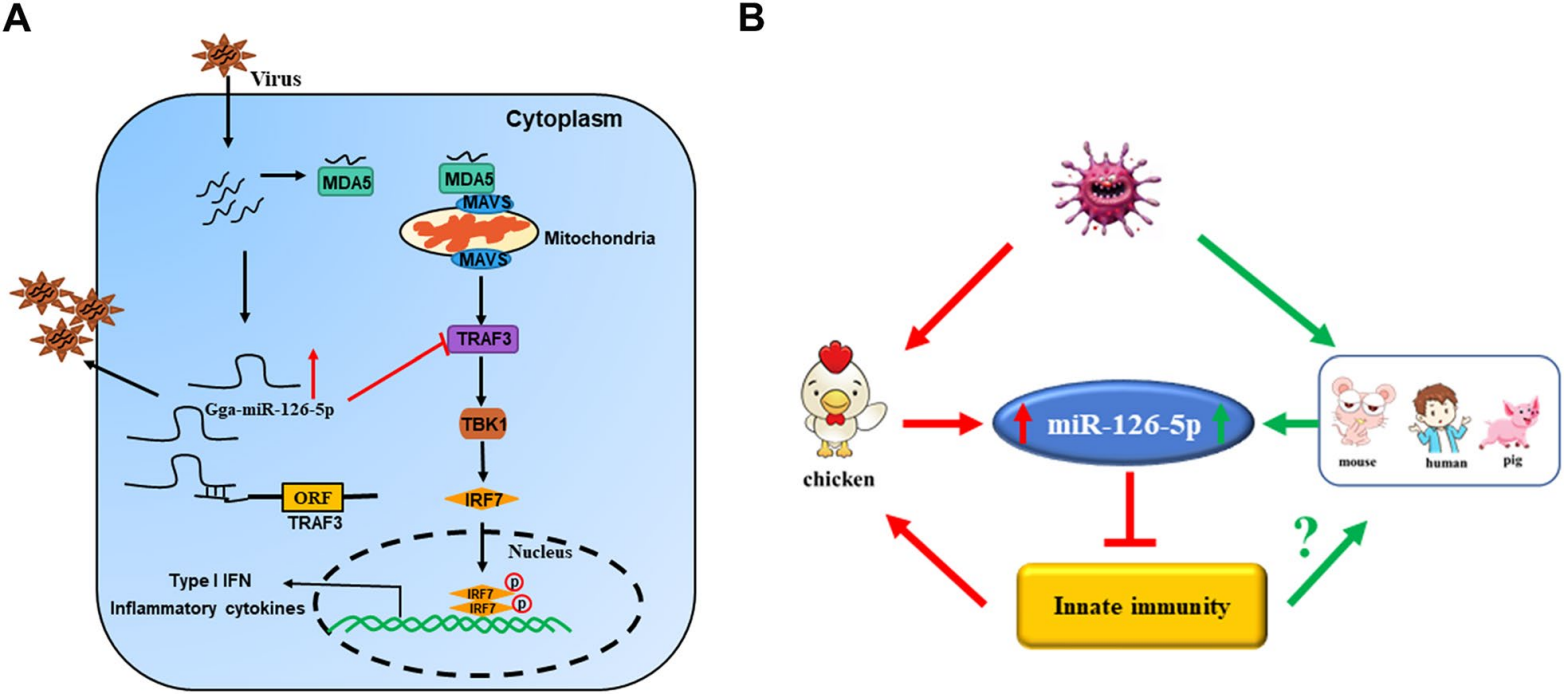
**Schematic illustration of miR-126-5p serving as an immune rheostat by targeting TRAF3.**

In conclusion, our research determined that miR-126-5p negatively regulates chicken antiviral innate immunity. After RNA virus infection, the expression of miR-126-5p is upregulated, and miR-126-5p inhibits the type I interferon signaling pathway and inflammatory factors by targeting TRAF3. We also found that miR-126-5p is widely present in different species, and influenza virus infection with A549 cells also changes the expression of miR-126-5p [48]. Therefore, it may also participate in the mammalian antiviral immune response. However, the specific role of miR-126-5p in mammalian innate immunity remains unclear. Therefore, our research will provide potential targets for the treatment of cytokine storms caused by viral infections (Figure 10).

## Supplementary Information

**Additional file 1. Primers used to detect relative gene mRNA expression levels with quantitative RT-PCR.**

**Additional file 2. Analysis of differential miRNA in chickens infected with Newcastle disease virus (NDV) or avian influenza virus (AIV).** (A) The Vene Map different miRNAs of chicken infection NDV or AIV. (B) The Heat Map differential miRNAs of chicken infection with NDV. (C) The Volcano Map different miRNAs of chicken infection with AIV. The miRNA microarray using the R package “limma”. |logFC|> 2 and a *p*-value < 0.05 were set as the threshold to screen out the different miRNAs.

## Data Availability

The data analyzed during the current study are available from the corresponding author upon reasonable request.

## Abbreviations

AIV: avian influenza virus
NDV: Newcastle disease virus
TRAF3: TNF receptor-associated factor 3
IFNβ: interferon-beta
PKR: eukaryotic translation initiation factor 2 alpha kinase 2
MX1: MX1–myxovirus (influenza virus) resistance 1
IL-1β: inflammatory cytokine related genes interleukin 1β
IL-6: interleukin 6
IL-8: interleukin-8
