# Supplementary material for: Chicken miR-126-5p negatively regulates antiviral innate immunity by targeting TRAF3
Source: Vet Res. 2022 Oct 12;53:82. doi: 10.1186/s13567-022-01098-x (PMC9559812; doi:10.1186/s13567-022-01098-x)
Supplement: Supplementary file 1 — Additional file 1. Primers used to detect relative gene mRNA expression levels with quantitative RT-PCR. [file 13567_2022_1098_MOESM1_ESM.docx]

**Additional file 1 Primers used to detect relative genes mRNA expression levels with quantitative RT-PCR.**

| **Genes** | **Primer** | **Nucleotide sequence of primers (5’-3’)** |
| --- | --- | --- |
| qchIFNβ | Forward | TCCTACTGCTCTTGCTTCTGC |
|  | Reverse | TGGAAATGGAAAAGTCACGTC |
| qchPKR | Forward | TGCTTGACTGGAAAGGCTACT |
|  | Reverse | TCAGTCAAGAATAAACCATGTGTG |
| qchMX1 | Forward | GTTTCGGACATGGGGAGTAA |
|  | Reverse | GCATACGATTTCTTCAACTTTGG |
| qchIL-1β | Forward | GCTCTACATGTCGTGTGTGATGAG |
|  | Reverse | TGTCGATGTCCCGCATGA |
| qchIL-6 | Forward | AGGACGAGATGTGCAAGAAG |
|  | Reverse | TGCTGTAGCACAGAGACTCG |
| qchIL-8 | Forward | ATTCAAGATGTGAAGCTGAC |
|  | Reverse | AGGATCTGCAATTAACATGAGG |
| qchTRAF3 | Forward | CTGATTGTCAGTTTGAAGAGC |
|  | Reverse | ATGACACACGGGCAGTCTGT |
| qNDV-NP | Forward | TGCAGCAATGGTACTCCGTT |
|  | Reverse | CCTTTGCTACCGTGACCCAT |
| β-actin | Forward | CAGACATCAGGGTGTGATGG |
|  | Reverse | TCAGGGGCTACTCTCAGCTC |
