# Supplementary material for: Chicken miR-126-5p negatively regulates antiviral innate immunity by targeting TRAF3
Source: Vet Res. 2022 Oct 12;53:82. doi: 10.1186/s13567-022-01098-x (PMC9559812; doi:10.1186/s13567-022-01098-x)
Supplement: Supplementary file 2 — Additional file 2. Analysis of differential miRNA in chickens infected with Newcastle disease virus (NDV) or avian influenza virus (AIV). (A) The Vene Map different miRNAs of chicken infection NDV or AIV. (B) The Heat Map differential miRNAs of chicken infection with NDV. (C) The Volcano Map different miRNAs of chicken infection with AIV. The miRNA microarray using the R package “limma”. |logFC|> 2 and a p-value < 0.05 were set as the threshold to screen out the different miRNAs. [file 13567_2022_1098_MOESM2_ESM.docx]

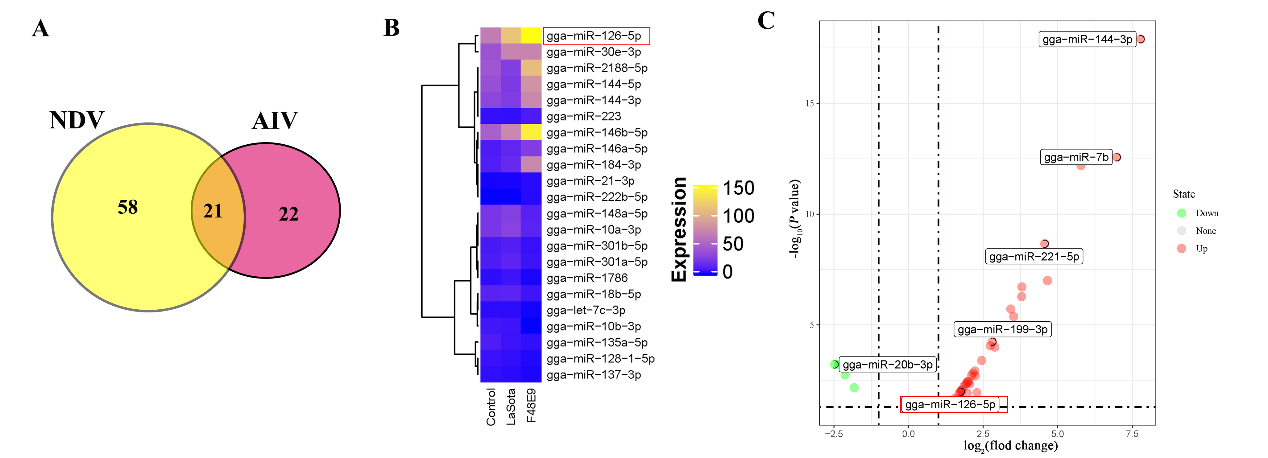


**Additional file 2 Analysis of differential miRNA in chickens infected with Newcastle disease virus (NDV) or avian influenza virus (AIV).** (A) The Vene Map different miRNAs of chicken infection NDV or AIV. (B) The Heat Map differential miRNAs of chicken infection with NDV. (C) The Volcano Map different miRNAs of chicken infection with AIV. The miRNA microarray using the R package “limma”. |logFC| >2 and a *p*-value <0.05 were set as the threshold to screen out the different miRNAs.
